# Synergistic Effects of Korean Red Ginseng Extract and the Conventional Systemic Therapeutics of Atopic Dermatitis in a Murine Model

**DOI:** 10.3390/nu14010133

**Published:** 2021-12-28

**Authors:** Yu Ri Woo, Seok Hoon Moon, Jeesuk Yu, Sang Hyun Cho

**Affiliations:** 1 Department of Dermatology, Incheon St. Mary’s Hospital, The Catholic University of Korea, Seoul 06591, Korea; w1206@naver.com; 2 Villa de Skin, Hwaseong 18478, Korea; windystep@naver.com; 3 Department of Pediatrics, Dankook University Hospital, Dankook University College of Medicine, Cheonan 31116, Korea; dryujs@dankook.ac.kr

**Keywords:** atopic dermatitis, antihistamine, cyclosporine, Korean Red ginseng, treatment

## Abstract

The synergistic effects of Korean Red ginseng (KRG, *Panax ginseng* C.A. Mey.) on conventional systemic therapeutics of atopic dermatitis (AD) have not been studied yet. To analyze the synergistic effects of KRG extract and the conventional systemic therapeutics of AD in TNCB-induced AD mouse model, we determined the change in modified scoring of index, the transepidermal water loss, the skin pathology, serum IgE, and the expression of various cytokines after combination treatment to the five-week-old NC/Nga female mice. The severity of AD was significantly decreased in the KRG + hydroxyzine (AH) group than AH group, and in the KRG + evening primrose oil (EPO) group than EPO group. A significant decrease in dermal inflammation was observed in the KRG + AH group than that in the AH group, and in the KRG + EPO group than that in the EPO group (*p* = 0.008), respectively. A decrease in CD1a expression was observed in the KRG + AH group when compared to the AH group (*p* = 0.008), and KRG + EPO group when compared to the EPO group. Compared to the CS group, the KRG + CS group showed a significant decrease in IL-17 expression. A combination of KRG and conventional systemic therapeutics can safely and effectively manage the AD.

## 1. Introduction

Atopic dermatitis (AD) is a very common cutaneous disorder. It is a relapsing and chronic cutaneous disease that significantly impairs the patient’s overall quality of life [1]. The pathophysiology of AD is not yet understood completely. However, various immunological dysregulations and dysfunctions of the epidermal barrier lead to initiation and aggravation of AD [2]. Previous studies have reported that the pathogenesis of AD is caused by an imbalance in the cytokine milieu of AD. In general, the abundant type 2 helper T cell (Th2)-mediated cytokines, such as interleukin (IL)-4, IL-5, and IL-13, play a pivotal role in the development and progression of AD [3]. At the initial stage of AD, pro-inflammatory cytokines, including IL-1, IL-6, and tumor necrosis factor (TNF)-α are produced by the inflammatory dendritic epidermal cells, which are different from recruited monocytes [4]. In addition, there is a shift to type 1 helper T cell-mediated (Th1) responses. This causes an increase in the expression of interferon (IFN)-γ [5]. This complex orchestration of inflammatory cytokines occurs in the pathogenesis of AD.

Due to its complicated pathogenesis, it is often difficult to treat and manage AD patients. In general, a combination of various treatment options is needed to treat AD patients. Mild AD cases are usually treated with only topical agents, whereas various systemic agents, such as systemic corticosteroids, cyclosporine A, antihistamines, and evening primrose oil are widely used to treat moderate to severe cases of AD [6,7,8].

Although researchers do not clearly know the exact the mechanism through which oral antihistamines benefit AD patients, they are widely prescribed by dermatologists for the management of AD. According to the Korean consensus guidelines for AD, oral antihistamines can be prescribed in AD patients to reduce pruritus and to inhibit scratching behavior in AD patients [9]. The common side effects of oral antihistamines are as follows: sedation, tachycardia, dry mouth, and blurred vision [9]. Systemic corticosteroids are well-known as systemic immunomodulatory agents that relieve the clinical symptoms of AD dramatically. However, dermatologists should avoid prescribing them on long-term basis because of the serious side effects [9]. Cyclosporine is another systemic immunomodulatory, which is used widely to manage moderate to severe cases of AD. This can be used relatively safely on patients who are older than 2 years [9]. However, the long-term use of cyclosporine could causes an increase in the risk of nephrotoxicity, hypercholesterolemia, osteoporosis, uncontrolled hypertension, and malignancy [10,11]. Therefore, there is a need for newer treatment strategies that are effective in AD while being safely used for a long-term period in AD.

*Panax ginseng* C.A. Meyer is cultivated in Korea. For thousands of years, this has been frequently consumed orally in the Far East region of Asia. In this region, people believe it to be a traditional medicine for a variety of inflammatory conditions. The Korean Red ginseng (KRG) is a type of Korean ginseng, which is harvested for six years. Then it is steamed and the dried root of *P. ginseng* is consumed. Currently, various studies have reported about how KRG exerts anti-inflammatory and anti-allergic effects on patients with different clinical conditions [12,13]. The major component of KRG is ginsenosides, which is a steroidal saponin with a distinct chemical structure [14]. The major active ingredient of KRG is ginsenosides, which contains an aglycone and a dammarane skeleton [15]. In our previous studies, we have described the therapeutic effects of KRG in a 2,4,6-trinitro-1-chlorobenzene (TNCB)-induced NC/Nga mice model of AD [16,17]. The KRG extract significantly improved the clinical symptoms of AD in NC/Nga mice. Moreover, it also helped in preventing the worsening of AD-like skin lesions in the animal model. When KRG was administrated systemically in mice, it significantly suppressed T-cell mediated pro-inflammatory cytokines. This led to a decrease in the expression levels of TSLP, TNF-α, IFN-γ, IL-31, and serum immunoglobulin E (IgE) [17]. In addition, it is relatively safe to systemically administer KRG in humans as it does not cause serious side effects [18,19].

Although the positive therapeutic effects of KRG have been reported in various previous studies, KRG extract tends to be added to conventional therapeutic medicines of AD. As not a single study has described the effect of the concomitant use of KRG extract with conventional systemic medications for AD. Therefore, this study focuses on deciphering the synergistic effects of KRG extract and the conventional treatments of AD, which were used concomitantly in the TNCB-induced AD mouse model. The synergistic effect of KRG extract was determined by assessing the clinical severity scores, the transepidermal water loss (TEWL), histological changes, serum IgE levels, and the expression profiles of various cytokines.

## 2. Materials and Methods

### 2.1. Animals

Five-week-old NC/Nga female mice were purchased from Charles River Laboratories (Kanagawa, Japan). There mice were housed in a specific pathogen-free environment at 22 °C for 7 days. The humidity was maintained at 50 ± 10% in the 12-h light and 12-h dark cycle. Sterilized food and water were provided to the mice. Animal care, handling, and experimental procedures were performed according to a protocol approved by the Animal Care and Use Committee of the Catholic University of Korea (CIMH 2017-008).

### 2.2. The Induction of AD-Like Skin Lesions

In this experiment, TNCB was purchased from Sigma-Aldrich Ltd. (St. Louis, MO, USA) and was used to induce AD-like skin lesions. Firstly, the abdomen of mice was sensitized epicutaneously with 150 μL of 5% TNCB solution, which was prepared in the solvent mixture of ethanol and acetone (4:1) on day 1. On day 5, the ears and dorsal skin of the mice were challenged with 150 μL of 1% TNCB solution, which was prepared in olive oil.

### 2.3. Animal Groups and Drugs

In this experiment, 45 NC/Nga mice were randomly segregated into following nine groups: Sham (negative control), AD (positive control), KRG, cyclosporine (CS), KRG + CS, hydroxyzine (AH), KRG + AH, evening primrose oil (EPO), and KRG + EPO groups. Each group consisted of five mice. The 5% TNCB sensitization and 1% TNCB challenge were performed on every experimental group, except the negative control group.

The KRG extract (Korea Ginseng Corporation, Daejeon, Korea) was suspended in distilled water. The KRG extract was manufactured from the roots of a 6-year-old fresh ginseng plant (*Panax ginseng* Meyer). The ginseng was harvested in the Republic of Korea by the farmers working for the Korea Ginseng Corporation (Seoul, Korea). When analyzed with high-performance liquid chromatography, resultant KRG extract was composed of ginsenosides Rb1 (5.85 mg/g), Rb2 (2.17 mg/g), Rc (2.29 mg/g), Rd (0.89 mg/g), Re (0.82 mg/g), Rf (1.37 mg/g), Rg1 (0.69 mg/g), Rg2s (1.50 mg/g), Rg3s (4.43 mg/g), Rg3r (2.02 mg/g) and Rh1 (1.28 mg/g).

In every group that received KRG, the mice were orally administered 200 mg/kg KRG extract by gastric intubation through an animal-feeding needle. Each mouse of the sham group and AD control group administrated with 0.2 mL phosphate-buffered saline (PBS), which was used as the control solution.

The mice from the experimental groups were orally administered following systemic therapeutics: 2.5 mg/kg cyclosporine (Cipol-N^®^, Chong Kun Dang, Seoul, Korea), 0.5 mg/kg hydroxyzine (Adipam^®^, Taiguk Pharm, Seoul, Korea), 50 mg/kg evening primrose oil (Evoprim^®^, Dalim Biotech, Seoul, Korea). These medications were administered daily with a gastric feeding tube for seven days, starting from the day of 5% TNCB sensitization.

### 2.4. The Measurement of the Severity of AD and the Transepidermal Water Loss

To evaluate the clinical severity of AD, we scored the clinical signs and symptoms of AD by using the modified scoring atopic dermatitis (SCORAD) index. As SCORAD index also assesses the subjective symptoms of AD, we used modified SCORAD index, which excludes the measurement of subjective symptoms of AD in this mouse model study. The modified SCORAD index measures the clinical severity of AD by calculating the sum of each score grades: 0 (none), 1 (mild), 2 (moderate), and 3 (severe) for each of the five manifestations: scaling/dryness, erythema/hemorrhage, excoriation/erosion, and edema. The assessment was performed by a blinded investigator. The skin barrier function was determined by measuring TEWL. We assessed TEWL on the dorsal skin of NC/Nga mice by using tewameter (Tewameter MPA5^®^, Courage & Khazawa, Köln, Germany). The TEWL of the same skin lesion of each mouse was measured three times with a consistent pressure of application by one blinded investigator in a conditioned room (25 °C with humidity of 50 ± 10%).

### 2.5. Skin Histology and Immunohistochemical Examination

On day 8, we performed 6 mm punch biopsies on the backs of mice. The excised back tissues were fixed in 4% paraformaldehyde. Then, these tissues were embedded in paraffin. Finally, 5 μm thin sections were cut with probe-on-plus slides (Fisher Scientific, Pittsburg, PA, USA). The de-paraffinized skin sections were stained with hematoxylin and eosin (H&E). To identify immunohistochemical markers, we stained some sections with monoclonal antibodies of TNF-α, IL-4, CD1a, IFN-γ, and IL-17. A high-temperature antigen unmasking technique was used to perform immunohistochemical analysis. The sections were heated in an unmasking solution (citrate buffer, pH 6.0). Then they were washed and incubated with mouse primary monoclonal antibodies at room temperature for one hour. After the completion of this procedure, the sections were incubated with secondary antibodies (Envision Detection kit K5007^®^, DAKO, Glostrup, Denmark). The reaction products were developed with diaminobenzidine solution, which was used as a chromogen. After the completion of chromogen reactions, we rinsed the sections and counterstained them with hematoxylin. Thereafter, the sections were dehydrated and covered with paramount (Fisher Scientific, Fair Lawn, NJ, USA). All the slides were visualized with a light microscope. To assess the histological changes, these slides were photographed and analyzed with imaging software (Cellsens^®^, Olympus, Tokyo, Japan). Two blinded investigators performed quantitative analysis of immunohistochemical examination: the stained cells were counted in a total of three random microscopic fields under the same magnification (400×). To measure epidermal thickness, the length of the cellular part of the epidermis from photographed slides was calibrated by imaging software (Cellsens^®^, Olympus, Tokyo, Japan).

Semi-quantitative analysis was also performed by two blinded investigators for assessing general histopathological characteristics including degree of hyperkeratosis, spongiosis, and dermal inflammation. Each investigators semi-quantitatively assessed all the sections and scored them using a five-point grading scale according to the severity of histological changes: 0, normal range or none; (1), slight; (2), moderate; (3), marked; and (4), very marked [20]. To measure epidermal thickness, the length of the cellular part of the epidermis from photographed slides was calibrated by imaging software (Cellsens^®^, Olympus, Tokyo, Japan).

### 2.6. Enzyme-Linked Immunosorbent Assay (ELISA)

Blood samples were collected from retro-orbital plexus of anesthetized mice and they were added to tubes coated with coagulation activator (Chase Scientific Glass, Inc., Rockwood, TN, USA). These samples were subjected to centrifugation and stored at −20 °C until further use. To determine serum concentrations of total IgE, we used a mouse IgE ELISA kit (Bethyl, Montgomery, TX, USA) according to manufacturer’s instructions.

### 2.7. Statistical Analysis

For comparison of differences between the controls and treatment groups were statistically determined by performing one-way analysis of variance (ANOVA) followed by a post hoc Tukey analysis was applied. In case of deviation from normal distribution, we conducted the Kruskal–Wallis followed by Dunn’s multiple comparison tests. All the statistical calculations were performed by using SPSS v21.0 software (SPSS Inc., Chicago, IL, USA). All the values were expressed as mean ± standard error of the mean (SEM). A *p*-value of less than 0.05 was considered to be statistically significant.

## 3. Results

### 3.1. The Clinical Effects of KRG and the Conventional Systemic Therapeutics of AD on the Skin TNCB-Induced AD Mouse Model

**Figure 1:**
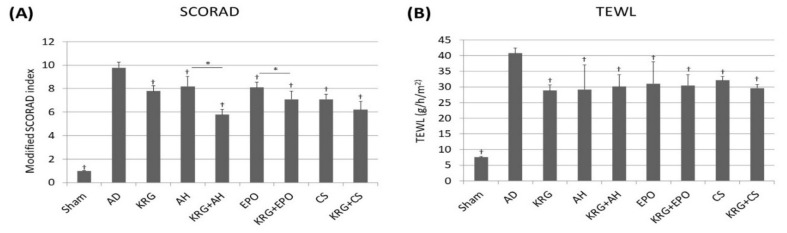
The clinical effects of KRG and the conventional systemic therapeutics of AD on the skin of NC/Nga mice (**A**) A modified SCORAD index for assessing the clinical severity of skin lesions in NC/Nga mice. (**B**) The changes in the epidermal barrier function were determined by measuring TEWL of the dorsal skin of the mice. † indicates *p* < 0.05 compared to the AD group. * indicates *p* < 0.05 in AH versus KRG + AH, CS versus KRG + CS, EPO versus KRG + EPO, respectively. All the data were presented as the mean and the standard error of the mean. Abbreviation: AD, atopic dermatitis; AH, antihistamine; CS, cyclosporine; EPO, evening primrose oil; KRG, Korean Red ginseng; SCORAD, scoring atopic dermatitis, TEWL, transepidermal water loss; TNCB, 2,4,6-trinitro-1-chlorobenzene.

Following sensitization and challenge of mice with TNCB, skin lesions of AD were produced in mice. To identify the changes in clinical severity of skin lesions, we measured the mean value for the modified SCORAD index and represented them in Figure 1A. The Kruskal–Wallis test showed that there was a significant difference in TEWL (*p* < 0.001) and SCORAD (*p* < 0.001) among groups. Compared to AD control group, the AH group, the KRG + AH group, the CS group, the KRG + CS group, the EPO group, and the KRG + EPO group showed a significantly decreased severity on the modified SCORAD index (*p* < 0.001), respectively on day 8. Moreover, the modified SCORAD score of KRG + AH group was significantly lower than that of AH group (*p* = 0.004). Furthermore, the modified SCORAD score of KRG + EPO group was significantly lower than that of EPO group (*p* = 0.044). All the statistical analysis was performed on day 8.

To determine the objective barrier function of skin, we measured TEWL. Figure 1B shows that compared to AD group, TEWL was found to be significantly lower in KRG group (*p* = 0.03), AH group (*p* = 0.029), KRG + AH group (*p* = 0.005), CS group (*p* = 0.031), KRG + CS group (*p* = 0.001), EPO group (*p* = 0.030), and KRG + EPO group (*p* = 0.007), respectively.

### 3.2. The Histopathological Changes Caused by the Synergistic Effects of KRG and Conventional Systemic Therapeutics of AD in TNCB-Induced AD Mouse Model

**Figure 2:**
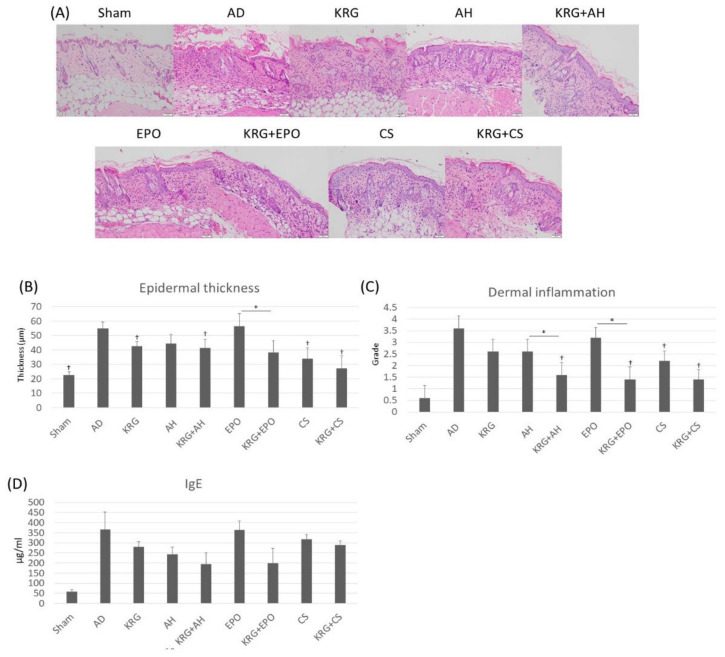
After the administration of KRG and conventional systemic therapeutics, the dermal inflammation and acanthosis was inhibited in TNCB-induced AD mouse model. (**A**) Representative images of histopathological analysis in each treatment group (H&E, 200x). Scale bar, 50 μm. (**B**) The bar graph of epidermal thickness in each group. (**C**) The bar graph of dermal inflammation in each group. (**D**) The bar graph of the serum total IgE in each group. † indicates *p* < 0.05 compared to the AD group. * indicates *p* < 0.05 in AH versus KRG + AH, CS versus KRG + CS, EPO versus KRG + EPO, respectively. All the data were presented as the mean and the standard error of the mean. Abbreviation: AD, atopic dermatitis; AH, antihistamine; CS, cyclosporine; EPO, evening primrose oil; KRG, Korean Red ginseng; TNCB, 2,4,6-trinitro-1-chlorobenzene.

The general histopathological patterns of groups were analyzed and summarized in Table 1 and Figure 2. The Kruskal–Wallis test indicated that there was a significant difference in degree of hyperkeratosis (*p* < 0.001), and spongiosis (*p* = 0.022) among groups. The ANOVA test showed a significant difference in the degree of dermal inflammation (*p* < 0.001) and epidermal thickness (*p* < 0.001) among groups. In AD group, hyperkeratosis, acanthosis, and dermal inflammation were significantly greater than those in the sham group (*p* < 0.001). Compared with the AD group, we observed a significant decrease in the epidermal thickness of the KRG group (*p* = 0.001), KRG + AH group (*p* = 0.001), CS group (*p* = 0.005), and KRG + CS group (*p* < 0.001; Figure 2B). Of note, the epidermal thickness of the KRG + EPO group was found to be significantly lesser than that of the EPO group (*p* = 0.021). Furthermore, the dermal inflammation of sham group (*p* < 0.001), KRG + AH group (*p* < 0.001), CS group (*p* = 0.004), KRG + CS group (*p* < 0.001), and KRG + EPO group (*p* < 0.001) was found to be significantly lesser than AD group (Figure 2C). In addition, the dermal inflammation of KRG + AH group was found to be significantly lesser than that of AH group (*p* = 0.019) on day 8. Likewise, the dermal inflammation of KRG + EPO group on day 8 was found to be significantly lesser than that of EPO group (*p* < 0.001).

### 3.3. Serum IgE Levels in TNCB-Induced AD Mouse Model That Was Administered KRG and Conventional Systemic Therapeutics of AD

To determine whether KRG and conventional systemic therapeutics of AD affected IgE-related responses, we measured serum IgE levels with an ELISA kit on day 8. There was a significant difference in serum IgE levels by Kruskal–Wallis test (*p* = 0.012) among groups. When compared to the AD group, total serum IgE levels were lower in KRG group, AH group, KRG + AH group, and KRG + EPO group; however these differences were not statistically significant (Figure 2D). Compared to the single treatment groups, the serum levels of IgE was lower in all the combination treatment groups (KRG + AH, KRG + EPO, and KRG + CS groups); however, these differences were not statistically significant.

### 3.4. The Changes in the Expression Profiles of Various Cytokines after the Administration of KRG and Conventional Systemic Treatments of AD in TNCB-Induced AD Mouse Model

**Figure 3:**
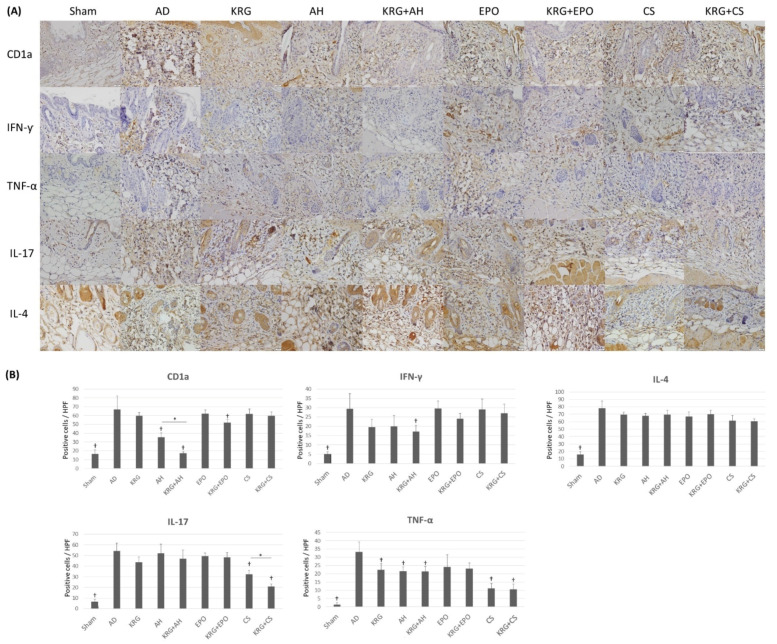
The expression profiles of various cytokines after administering KRG and conventional systemic therapeutics of AD in TNCB-induced AD mouse model (**A**) The representative images of immunohistochemical stainings of skin lesions in each group (CD1a, 400x; IFN-γ, 400x; TNF-α, 400x; IL-17, 400x; IL-4, 400x). Scale bar, 20 μm. (**B**) The bar graphs illustrate the quantification of CD1a, IFN-γ, TNF-α, IL-17, and IL-4 expressions in each group. † indicates *p* < 0.05 compared to the AD group. * indicates *p* < 0.05 in AH versus KRG + AH, CS versus KRG + CS, EPO versus KRG + EPO, respectively. All the data were presented as the mean and the standard error of the mean. Abbreviation: AD, atopic dermatitis; AH, antihistamine; CS, cyclosporine; EPO, evening primrose oil; KRG, Korean Red ginseng.

The changes in the expression profiles of various cytokines of AD were determined on day 8. There was a significant difference in degree of expression of CD1a (*p* < 0.001), TNF-α (*p* < 0.001), IL-17 (*p* < 0.001), and IL-4 (*p* < 0.001) by Kruskal–Wallis test among groups. The ANOVA test showed a significant difference in the expression of IFN-γ (*p* < 0.001) among groups. Compared to the AD group, there was a significant decrease in the expression of CD1a in sham group (*p* < 0.001), AH group (*p* = 0.006), KRG + AH group (*p* < 0.001), and KRG + EPO group (*p* = 0.024; Figure 3). Compared to the AH group, the KRG + AH group showed a synergistic effect in decreasing the expression of CD1a (*p* = 0.048). Furthermore, compared to the AD group, the KRG + AH group showed a significantly lower expression of IFN-γ (*p* = 0.008). When compared to the AD group, the expression of TNF-α was also found to be lower in sham group (*p* < 0.001), KRG group (*p* = 0.048), AH group (*p* = 0.049), KRG + AH group (*p* = 0.048), CS group (*p* = 0.008), and KRG + CS group (*p* = 0.006). In addition, the expression of IL-17 was observed to be lower in sham group (*p* < 0.001), CS group (*p* < 0.001) and KRG + CS group (*p* < 0.001) as compared to that in AD group. Notably, compared to CS group, the KRG + CS group also showed a synergistic effect in decreasing the expression of IL-17 (*p* = 0.046).

## 4. Discussion

In this study, we orally administered KRG and remarkably improved the skin lesions of AD in TNCB-induced AD mouse model. Furthermore, we also found that the synergistic effects of KRG and conventional systemic treatments of AD were beneficial in improving the clinical severity of AD. Moreover, histopathological analysis also revealed a significant decrease in the dermal inflammation of experimental mice, which were treated with both KRG and conventional systemic therapeutics of AD.

The oral administration of KRG extract showed clinical efficacy in AD by reducing the scratching behaviors, increasing the TEWL, and decreasing dermal inflammation in a murine model study [16,17], which is in line with findings from this study. Moreover, the expression of TNF-α was suppressed after administration of KRG extract [17], which is also in agreement with the findings of this study. To further clarity the synergistic effects of KRG and the conventional systemic therapeutics of AD, we compared the effects of the combined administration of KRG and conventional systemic therapeutics of AD in this study. Among the various conventional systemic therapeutics of AD, we only determined the synergistic effects of cyclosporine, antihistamine, and evening primrose oil. This is because these medications are widely used in managing AD for a long-term period.

Cyclosporine is an immunosuppressant that exerts its action on T cells by inhibiting the activity of T cells via several mechanisms [21]. Among them, cyclosporine suppresses the T cell activation mediated by the presentation of antigens through blocking the downstream singling pathway of the T-cell receptor [11]. In various clinical trials, it has been found that patients with AD have benefitted immensely from cyclosporine [22,23]. Compared to the AD control group, the group treated with a combination of KRG and cyclosporine showed a significant decrease in the expression of IL-17 and TNF-α. Notably, a significant synergistic effect of KRG and cyclosporine elicited a decline in the expression of IL-17. In the group treated with only KRG, there was no significant decrease in the expression of IL-17. In the group treated with a combination of cyclosporine and KRG, the decrease in the expression of IL-17 was significant as compared to that observed in the group treated with only cyclosporine. Recently, several studies have reported about the important role played by Th17 cytokines in AD. In one study, researchers found that an increased number of IL-17 positive cells infiltrated in the dermal skin of patients with AD [24]. Moreover, an increased expression of Th17 gene and a decreased expression of Th1 gene were observed in some Asian patients with AD [25]. This indicates that the expression of IL-17 in particular subtypes of AD could be observed and inhibiting the IL-17 expression might be promising option in some AD phenotypes managing AD phenotypes. Although additional studies must be conducted to understand the mechanism of action of these combined therapies in AD, we propose that the concomitant use of cyclosporine and KRG is beneficial in reducing the inflammation caused by the expression of IL-17.

Findings from these results suggest that antipruritic and anti-inflammatory effect of KRG may potentiate the anti-inflammatory effect of cyclosporine, and influence the decreasing clinical severity. Although cyclosporine is effective in managing AD, it is a potent immunosuppressant that causes many side effects. Therefore, we decided to achieve a similar effect with a smaller dose of cyclosporine and KRG. With this strategy, we reduced the side effects of cyclosporine, but maintained its therapeutic effect on patients with AD.

In this study, we also determined the synergistic effect of KRG extract and antihistamine. Using the modified SCORAD index, we found that compared to the group treated with only antihistamine, the group treated with a combination of KRG extract and antihistamine showed a clinically significant improvement in AD. Compared to the group treated with only antihistamine, the group treated with combination of KRG and antihistamine was found to have a significantly lower dermal inflammation. Previous studies also found that the expression of CD1a positive cells was reduced after administering the mice with only KRG extract [17,26]. This finding is in complete agreement with this study. In this study, compared to the group treated with only antihistamine, the group treated with a combination of KRG and antihistamine showed a significant decrease in dermal expression of CD1a. Although researchers still do not completely understand how CD1a positive cells are involved in the pathogenesis of AD, it is now clear that an increased expression of CD1a is related to skin lesions of AD [27]. This implies that CD1a positive cells can cause microenvironmental changes that lead to the development of AD. As increased expression of CD1a and CD1a positive antigen presenting cells are associated with promoting the skin inflammation [28], a significant decreased expression of CD1a after combined administration of KRG and antihistamine suggests that their combination therapy is effective in suppressing the early stage of inflammation. The antihistamines are quite effective in relieving pruritus in patients with AD [29]. In our previous study, the scratching behavior of a murine model of AD decreased following the administration of KRG [16], suggesting the anti-pruritic effect of KRG in AD. In summary, we suppose that a significant synergistic effect might be observed when patients with AD are treated with a combination of antihistamine and KRG through potentiating antipruritic effect of two agents and regulating the earlier phase of inflammation. Antihistamine is a relatively safe treatment and has few serious side effects. Thus, the combinational treatment of antihistamine and KRG could be an attractive treatment options in AD as it reduces skin inflammation and improves clinical symptoms of AD.

Currently, EPO has been prescribed as an adjunctive treatment for patients with AD. This is because EPO is rich in essential fatty acids, such as gamma-linoleic acid. It is relatively safe to include dietary supplements of EPO because it has few side effects. Thus, it is preferred mode of treatment for patients with AD. Following the administration of EPO, patients with AD are relieved of skin dryness and itching [30]. Since EPO is effective in controlling various chronic inflammatory conditions by altering the metabolism of eicosanoid [31], the administration of EPO could be a beneficial in normalizing the altered epidermal eicosanoid metabolism in the lesional epidermal skin of AD patients [32]. Although researchers have not yet elucidated whether KRG plays a positive role in regulating epidermal eicosanoid metabolism, they do know that KRG possibly controls eicosanoid biosynthesis in macrophages, platelets, and vascular smooth muscles [33,34]. In this study, we found that the group treated with both KRG and EPO showed a significant decrease in the clinical severity of AD as compared to the group administered with only EPO. Notably, the group treated with a combination of KRG and EPO exhibited a significant decrease in epidermal hyperplasia than the group administered with only EPO. Although further studies are needed to clarify this association, we have established that both KRG and EPO are involved in the metabolism of eicosanoid simultaneously. Therefore, both EPO and KRG extract should be administered to ensure a synergistic clinical effect in AD. The combined administration of EPO and KRG extract would probably modify the metabolism of eicosanoid and restore the skin barrier.

In conclusion, our results suggest that combined administration of KRG exhibited the significant synergistic effects with conventional systemic therapeutics of AD including cyclosporine, antihistamine, and EPO. We proved that the concomitant use of KRG and conventional systemic therapeutics of AD might be effective in alleviating the skin lesions of AD, while reducing serious side effects of conventional systemic therapeutics. We expect that findings from the present study could provide the potentials that combined use of KRG with conventional systemic therapeutics of AD could serve as an effective and safe therapeutic strategy in AD.

## Figures and Tables

**Table 1:** A comparison of general histopathological patterns.

Hematoxylin and Eosin	Sham	AD Control	KRG	AH	AH + KRG	EPO	EPO + KRG	CS	CS + KRG
**Epidermal change**									
Epidermal thickness	22.61 ± 2.21	54.81 ± 4.60	42.54 ± 3.16	44.36 ± 6.50	41.46 ± 5.84	56.49 ± 8.66	38.31 ± 8.06	34.0 ± 7.39	27.17 ± 8.93
Hyperkeratosis	0.20 ± 0.44	2.60 ± 0.54	1.40 ± 0.54	1.40 ± 0.54	1.20 ± 0.44	2.00 ± 0.70	1.40 ± 0.54	2.40 ± 0.54	2.40 ± 0.54
Spongiosis	0.00 ± 0.00	1.80 ± 0.44	1.40 ± 0.54	1.40 ± 0.54	1.40 ± 0.54	1.60 ± 0.54	1.40 ± 0.54	1.60 ± 0.54	1.60 ± 0.54
**Dermal change**									
Dermal inflammation	0.61 ± 0.54	3.60 ± 0.54	2.60 ± 0.54	2.60 ± 0.54	1.40 ± 0.54	3.20 ± 0.44	3.00 ± 0.54	2.20 ± 0.44	1.40 ± 0.54

Quantitative analysis for the epidermal thickness (μm) and semiquantitative analysis for the hyperkeratosis, spongiosis and dermal inflammation using the numerical grading system as follows: 0, none or normal range; (1), slight; (2), moderate; (3), marked; (4), very marked. The data are presented as mean ± standard error of the mean. Abbreviation: AD, atopic dermatitis; AH, antihistamine; CS, cyclosporine; EPO, evening primrose oil; KRG, Korean red ginseng.
